# Evaluation of therapeutic effect and prognostic value of ^18^F-FDG PET/CT in different treatment nodes of DLBCL patients

**DOI:** 10.1186/s13550-024-01074-w

**Published:** 2024-02-19

**Authors:** Wenyu Zhao, Xiaodong Wu, Shuo Huang, Hui Wang, Hongliang Fu

**Affiliations:** 1 Department of Nuclear Medicine, Xinhua Hospital, Shanghai Jiao Tong University School of Medicine, Shanghai, 200092 China; 2 Department of Radiology, Shandong Cancer Hospital and Institute, Shandong First Medical University & Shandong Academy of Medical Sciences, Jinan, 250117 Shandong China

**Keywords:** DLBCL, R/R, PET/CT, Semi-quantitative parameters, Visual analysis, Survival

## Abstract

**Background:**

In the present study, we aimed to investigate the role of baseline (B), interim (I) and end-of-treatment (Eot) ^18^F-FDG PET/CT in assessing the prognosis of diffuse large B cell lymphoma (DLBCL), so as to identify patients who need intensive treatment at an early stage.

**Methods:**

A total of 127 DLBCL patients (62 men; 65 women; median age 62 years) were retrospectively analyzed in this study. Baseline (*n* = 127), interim (*n* = 127, after 3–4 cycles) and end-of-treatment (*n* = 53, after 6–8 cycles) PET/CT images were re-evaluated; semi-quantitative parameters such as maximum standardized uptake value of lesion-to-liver ratio (SUVmax_(LLR)_) and lesion-to-mediastinum ratio (SUVmax_(LMR)_), total metabolic tumor volume (TMTV) and total metabolic tumor volume (TLG) were recorded. ΔTLG^1^ was the change of interim relative to baseline TLG (I to B), ΔTLG^2^ (Eot to B). ΔSUVmax and ΔTMTV were the same algorithm. The visual Deauville 5-point scale (D-5PS) has been adopted as the major criterion for PET evaluation. Visual analysis (VA) and semi-quantitative parameters were assessed for the ability to predict progression-free survival (PFS) and overall survival (OS) by using Kaplan–Meier method, cox regression and logistic regression analysis. When visual and semi-quantitative analysis are combined, the result is only positive if both are positive.

**Results:**

At a median follow-up of 34 months, the median PFS and OS were 20 and 32 months. The survival curve analysis showed that advanced stage and IPI score with poor prognosis, ΔSUVmax_(LLR)_^1^ < 89.2%, ΔTMTV^1^ < 91.8% and ΔTLG^1^ < 98.8%, ΔSUVmax_(LLR)_^2^ < 86.4% were significantly related to the shortening of PFS in patient (*p* < 0.05). ΔSUVmax_(LLR)_^1^ < 83.2% and ΔTLG^1^ < 97.6% were significantly correlated with the shortening of OS in patients (*p* < 0.05). Visual analysis showed that incomplete metabolic remission at I-PET and Eot-PET increased the risk of progress and death. In terms of predicting recurrence by I-PET, the combination of visual and semi-quantitative parameters showed higher positive predictive value (PPV) and specificity than a single index.

**Conclusion:**

Three to four cycles of R-CHOP treatment may be a time point for early prediction of early recurrence/refractory (R/R) patients and active preemptive treatment. Combined visual analysis with semi-quantitative parameters of ^18^F-FDG PET/CT at interim can improve prognostic accuracy and may allow for more precise screening of patients requiring early intensive therapy.

**Supplementary Information:**

The online version contains supplementary material available at 10.1186/s13550-024-01074-w.

## Introduction

DLBCL is the most common type of non-Hodgkin lymphoma (NHL) [1, 2], its malignant degree is high, and its proliferation rate is fast. Over the past 20 years, rituximab, cyclophosphamide, doxorubicin, vincristine and prednisone (R-CHOP) have been established as standard treatment options for DLBCL patients. Under current R-CHOP first-line immunochemotherapy, 80% of low-risk DLBCL patients (IPI:0–1) can achieve long-term remission, while 30% of medium-/high-risk patients (IPI:2–5) cannot. As for second-line treatment, the standard of care for chemo-sensitive patients with relapsed DLBCL is autologous stem-cell transplantation (ASCT). Approximately 25% of patients have response to salvage therapy, subsequently undergo ASCT and achieve durable remission. However, the prognosis for patients with early relapsed or refractory DLBCL remains poor [3]. Xu et al. [4] pointed out that, in second-line therapy, patients with early relapsed or refractory DLBCL have increased survival benefit from chimeric antigen receptor T cell (CAR-T) therapy. Therefore, it is necessary to predict early relapsed or refractory patients for preemptive CAR-T therapy. These patients are not easily identified by current clinical indices of risk, such as international prognostic indices (IPI) for post-treatment assessment.

^18^F-FDG PET/CT, as a commonly used imaging evaluation method in the diagnosis and judgment of DLBCL, has been widely used in staging, efficacy monitoring and prognosis evaluation [5]. Response evaluation is a key issue in defining the best treatment strategy for lymphoma patients. PET interpreted according to visual criteria is a matter of debate in DLBCL. Although some studies confirm the predictive value of visual dichotomy evaluation based on Deauville criteria [6], it is insufficient to guide treatment decision-making. Some studies have pointed out that the PET/CT parameters after middle and first-line chemotherapy can be used to semi-quantitatively evaluate the efficacy and prognosis of DLBCL. Maximum standard uptake value (SUVmax) is the most widely studied semi-quantitative index for reflecting the metabolic activity in lymphoma [7]. The cutoff for PET positive was increased from the level of mediastinal blood pool in the Cheson 2007 criteria to that of the liver background in the Lugano 2014 criteria [8]. Thus, in order to make a more individualized assessment of prognosis, the ratio of SUVmax between the lesion and the liver (lesion-to-liver SUV_max_ ratio, abbreviated as SUVmax_(LLR)_ will be used in this study. However, reliability of SUVmax is affected by partial volume effect, blood glucose level and time after injection [9]. Metabolic tumor volume (MTV) is a metabolic parameter of PET/CT based on the size of tumor focus. Currently, MTV has been shown to predict PFS of DLBCL [10]. At baseline PET scan, high MTV indicates poor prognosis [11]. Total lesion glycolysis (TLG) is the product of tumor MTV and SUVmean, which not only considers the metabolic volume of tumor, but also evaluates the level of glucose metabolism of tumor, which is closer to the concept of tumor load. TLG is of higher value in predicting the prognosis of DLBCL than SUVmax and MTV [12–14]. In the past 2 years, only a few clinical studies have explored the effects of changes in interim PET compared with baseline PET on prognosis, including ΔSUVmax, ΔMTV and Δ TLG, interim PET changes may be more accurate than baseline PET parameters in predicting prognosis. The prognostic effect of PET/CT after end of treatment is even less discussed. The utility of PET/CT in assessing response after end of treatment has been confirmed in several studies [15–17]. Previous studies have found interim PET was inferior to end-of-treatment PET for prognosis prediction [18, 19]. At present, the predictive value of each method remains subject to debate [20, 21]. The majority of studies have demonstrated that in patients with DLBCL, the PPV value predicted by semi-quantitative PET/CT scanning parameters is greater than that of visual analysis [22, 23]. In Gyorke et al. [24], a ΔSUV 48.9% cutoff was combined with D5-PS, which had a greater PPV value than using just one index. Thus, it is important to continue researching whether semi-quantitative and visual analysis can be used to increase the accuracy of PET/CT in evaluating the prognosis of DLBCL.

Consequently, the objective of this study was to evaluate how to find DLBCL patients with poor prognosis timely and accurately. That is, we retrospectively assessed the predictive value of semi-quantitative parameters (ΔSUVmax_(LLR)_, ΔTMTV, ΔTLG) and treatment metabolic response of ^18^FDG-PET/CT at baseline, interim and end of treatment for PFS and OS of DLBCL. And we investigated whether combining the two can improve the accuracy of predicting prognosis in DLBCL patients, as well as guide clinical treatment decisions.

## Patients and methods

### Patient population

A retrospective analysis was performed in the present study, which consisted of between May 2012 and December 2022. Inclusion criteria: (1) age ≥ 18 years, (2) histologically confirmed as DLBCL, (3) patients who underwent baseline PET/CT (B-PET/CT) and interim PET/CT (I-PET) after 3–4 cycles of chemotherapy, or/and end-of-treatment PET/CT (Eot-PET/CT) after all planned first-line therapy. Surgical resection and complicated with other tumors were exclude. Clinical pathological features of patients were also determined, including epidemiological features (gender, age), clinical information [IPI score, D-5PS scores, LDH (lactate dehydrogenase) level, β2-MG(Beta-2-microglobulin) level, ferritin level, Ann Arbor stage, pathological classification. Of the overall patients, all patients received R-CHOP or R-CHOP-like regimens.

### ^18^F-FDG PET/CT scan

^18^F-FDG PET/CT scan was performed on a Biograph 64 system (Siemens Healthineers, Erlangen, Germany) with a 21.6 cm axial field of view. Patients were required to fast for at least 6 h prior to imaging, and serum glucose levels were kept less than 7.4 mmol/l. Images were obtained about 60 min after intravenous administration of 3.7 MBq of ^18^F-FDG per kilogram of body weight. Six or seven bed positions from the base of the skull to the mid-thighs were captured. PET images were acquired for 2.5 min per bed position. CT was performed on the same scanner without contrast administration, and CT scan data were collected under the following conditions: 120 kV, 101 mA (adjusted by auto mA) and a gantry rotation speed of 0.5 s. All the CT scans were conducted via 5-mm-thick axial slices. PET images were reconstructed at 200 × 200 pixels using a Gaussian filter of 5.0 mm full width at half maximum value. All image reconstructions were performed with the ordered-subset expectation maximization algorithm, incorporating a CT-based transmission map.

### Semi-quantitative analysis

PET/CT imaging results were analyzed and interpreted by two experienced nuclear medicine physicians who were unaware of the patients’ clinical information, other conventional imaging findings and pathology results. The SUVmax, TMTV and TLG of systemic lesions in patients with DLBCL were measured by software Metavol (Hokkaido University, Sapporo, Japan) (Additional file 1: Fig. S1). A semi-automated tumor/non-tumor separation algorithm is implemented by a stepwise thresholding technique, that is, tumor/non-tumor was automatically separated by software operation when SUVmax > 2.5 by manually selecting tumor lesions, then the SUVmax, TMTV and TLG can calculate automatically. It can make more reproducible between operators [25, 26].

In baseline PET, the highest FDG uptake was considered to be the SUVmax of the patient. For the interim PET/CT and end-of-treatment PET/CT images, SUVmax was measured in residual lesions. If the lesion was disappeared after treatment, a region of interest was drawn in the same area on the baseline PET. In order to ensure the accuracy of the data, the two nuclear medicine doctors used the same standard to draw independently. After the data collection was completed, the two doctors carefully compared the data and re-checked the data when disagreements occurred. ΔSUVmax^1^ was the percentage change of SUVmax of I-PET compared to B-PET, ΔSUVmax^2^ was the percentage change of SUVmax of Eot-PET compared to B-PET; algorithms of the percentage change of ΔSUVmax, ΔTMTV and ΔTLG are the same.

### Evaluate of curved effect

The PET/CT results were assessed according to the D-5PS [27] criteria. The D-5PS scoring system was used to qualitatively evaluate the treatment response as follows [28]: (1) no uptake; (2) uptake ≤ mediastinal blood pool; (3) uptake > mediastinal blood pool; (4) uptake moderately increase compared with the liver uptake at any site; (5) uptake markedly increased compared with the liver at The D-5PS scoring system was used to qualitatively evaluate the treatment response as follows: (1) no uptake; (2) uptake ≤ mediastinal blood pool; (3) uptake > mediastinal blood pool, but ≤ liver; (4) uptake moderately increased compared with the liver uptake at any site; (5) uptake markedly increased compared with the liver at any site [21]. Scores of 4–5 were considered positive, while scores of 1–3 were considered negative.

### Follow-up

Patients were followed up by telephone or imaging data from May 2012 to December 2022. OS is defined as the time from the patient’s pathological diagnosis to death or the end of follow-up. PFS is defined as the time between the patient’s pathological diagnosis and the first discovery of tumor recurrence, progression, death or the end of follow-up. At a median follow-up of 34 months, the median PFS and OS were 20 and 32 months.

### Statistical analysis

All statistical analyses were performed using SPSS software (version 26.0). ^18^F-FDG PET/CT semi-quantitative parameters of ΔSUVmax_(LLR)_, ΔTMTV and ΔTLG, and clinicopathological findings were analyzed and compared by correlated analysis, the independent sample Wilcoxon rank sum test, or one-way ANOVA. Kaplan–Meier survival analysis was performed to predict PFS and OS. The predictive value of PET/CT semi-quantitative parameters and clinicopathological factors were analyzed via univariate and multivariate cox proportional hazards regression. The predictive value of visual assessment also was analyzed via logistic regression analysis. *p* < 0.05 indicated statistically significant data.

## Results

### Patient’s characteristics

A total of 127 patients with DLBCL included 62 men and 65 women. Among them, 14 patients had poor progress or poor curative effect in interim evaluation, and the second-line treatment was replaced later. Due to various reasons, only 53 patients continued to complete Eot-PET/CT follow-up. For patients who did not have Eot-PET/CT examination, we also followed up by telephone and other examinations. The median age was 62 years ranging from 23 to 84 years. The basic characteristics of the enrolled patients are shown in Table 1.

**Table 1:** Basic characteristics of clinical data of newly diagnosed DLBCL patients and their correlation with PET/CT metabolic parameters

Characteristic	Total population N = 127		SUVmax (median)	*p* value	TMTV (median)	*p* value	TLG (median)	*p* value
	No	%						
Age (years)				0.184		0.802		0.944
≤ 60	46	36.2	26.77	–	363.87	–	2738.12	–
> 60	81	63.8	29.98	–	188.51	–	1631.10	–
Gender	62	48.8		0.350		0.481		0.309
Female	65	51.2	30.70	–	269.87	–	1961.89	–
Male	62	48.8	28.18	–	206.27	–	1760.97	–
Stages				0.343		0.000***		0.000***
I-II	45	35.4	39.04	–	84.50	–	709.31	–
III-IV	82	64.6	29.69	–	419.06	–	3273.09	–
COO subtypes				0.805		0.834		0.844
GCB	33	28.9	29.04	–	337.50	–	3268.23	–
Non-GCB	81	71.7	29.87	–	193.68	–	1631.10	–
IPI score				0.950		0.000***		0.000***
0–2	56	44.1	29.63	–	102.41	–	832.09	–
3–5	71	55.9	29.07	–	420.60	–	3277.96	–
LDH				0.164		0.000***		0.000***
Normal	54	42.5	27.70	–	84.50	–	604.14	–
Elevated	73	57.5	30.48	–	419.56	–	3734.96	–
β2-MG				0.145		0.000***		0.000***
Normal	54	42.5	26.12	–	110.09	–	947.81	–
Elevated	73	57.5	30.64	–	420.60	–	3277.96	–
Ferritin				0.547		0.013*		0.014*
Normal	77	60.6	29.42	–	151.18	–	1460.10	–
Elevated	50	39.4	29.26	–	387.29	–	2741.69	–

***p < 0.001, **p < 0.01, *p < 0.05

*DLBCL* diffuse large B cell lymphoma, PET/CT positron emission tomography/computed tomography, *COO* cell of origin, *GCB* germinal center B cell, *IPI* international prognostic index, *LDH* lactate dehydrogenase, *β2-MG* β2-microglobulin, *SUVmax* maximum standardized uptake value, *TMTV* total metabolic tumor volume, *TLG* total lesion glycolysis

Univariate cox regression analysis of PFS showed that advanced stage and IPI score with poor prognosis were associated with PFS (*p* < 0.05, Table 3). However, univariate cox regression analysis showed that all clinical parameters had no significant difference in poor OS (*p* > 0.05, Table 4).

### Semi-quantitative parameters’

#### Baseline PET/CT

##### Correlation between clinical characteristics of patients in relation to semi-quantitative parameters

**Fig. 1:**
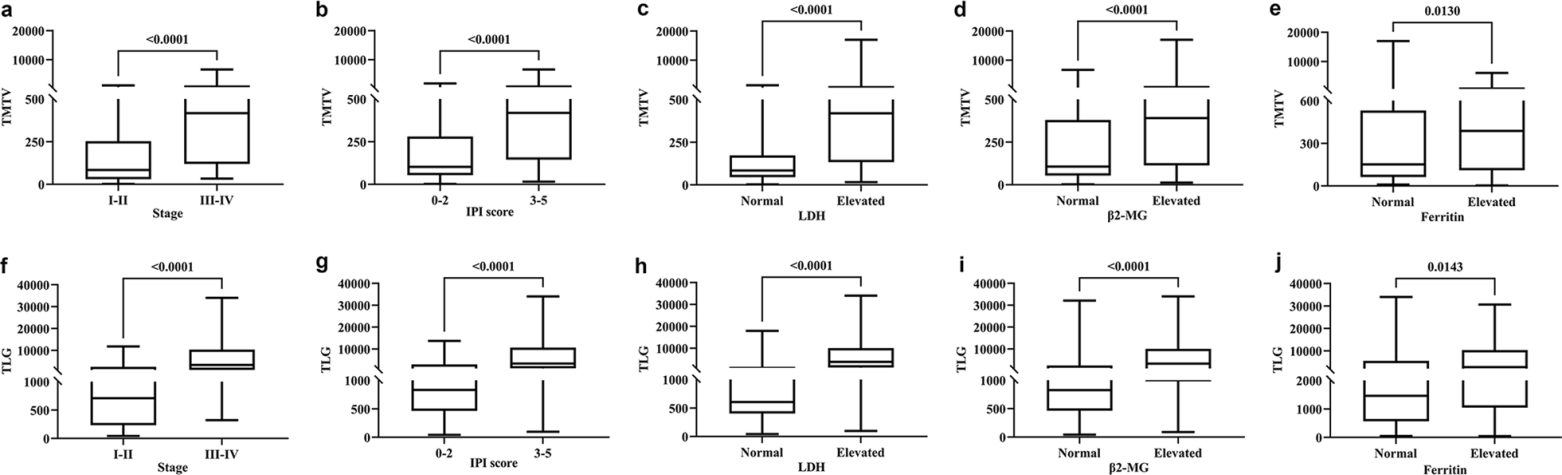
The correlation between Ann Arbor stage (**a**&**f**, *p* < 0.05), IPI score (**b**&**g**,* p* < 0.05), LDH (c&h, *p* < 0.05), β2-MG (**d**&**i**,* p* < 0.05), ferritin (**e**&**j**,* p* < 0.05) and TMTV&TLG by an independent sample rank sum test, respectively

Independent-sample Wilcoxon rank sum test showed that advanced Ann Arbor stage, IPI score of poor prognostic and elevated LDH, β2-MG and ferritin were positively and significantly associated with high TMTV and TLG (*p* < 0.05) (Table 1, Fig. 1).

Spearman rank correlation analysis showed high levels of LDH, β2-MG and ferritin were positively and significantly associated with high TMTV and TLG (*p* < 0.05) (Table 2).

**Table 2:** Correlation between baseline clinic characteristic and semi-quantitative parameters

clinic characteristic	semi-quantitative parameters					
	SUVmax		TMTV		TLG	
	*r* value	*p* value	*r* value	*p* value	*r* value	*p* value
LDH	0.056	0.537	0.690	0.000***	0.666	0.000***
β2-MG	0.053	0.569	0.463	0.000***	0.438	0.000***
Ferritin	0.065	0.508	0.324	0.001**	0.300	0.002**

***p < 0.001, **p < 0.01, *p < 0.05

*LDH* lactate dehydrogenase, *β2-MG* β2-Microglobulin, *SUVmax* maximum standardized uptake value, *TMTV* total metabolic tumor volume, *TLG* total lesion glycolysis

#### Interim PET/CT (I-PET)

All the patients (127) underwent I-PET after 3–4 cycles of chemotherapy (median of four cycles). At a median follow-up of 34 months, the median PFS and OS were 20 and 32 months.

**Fig. 2:**
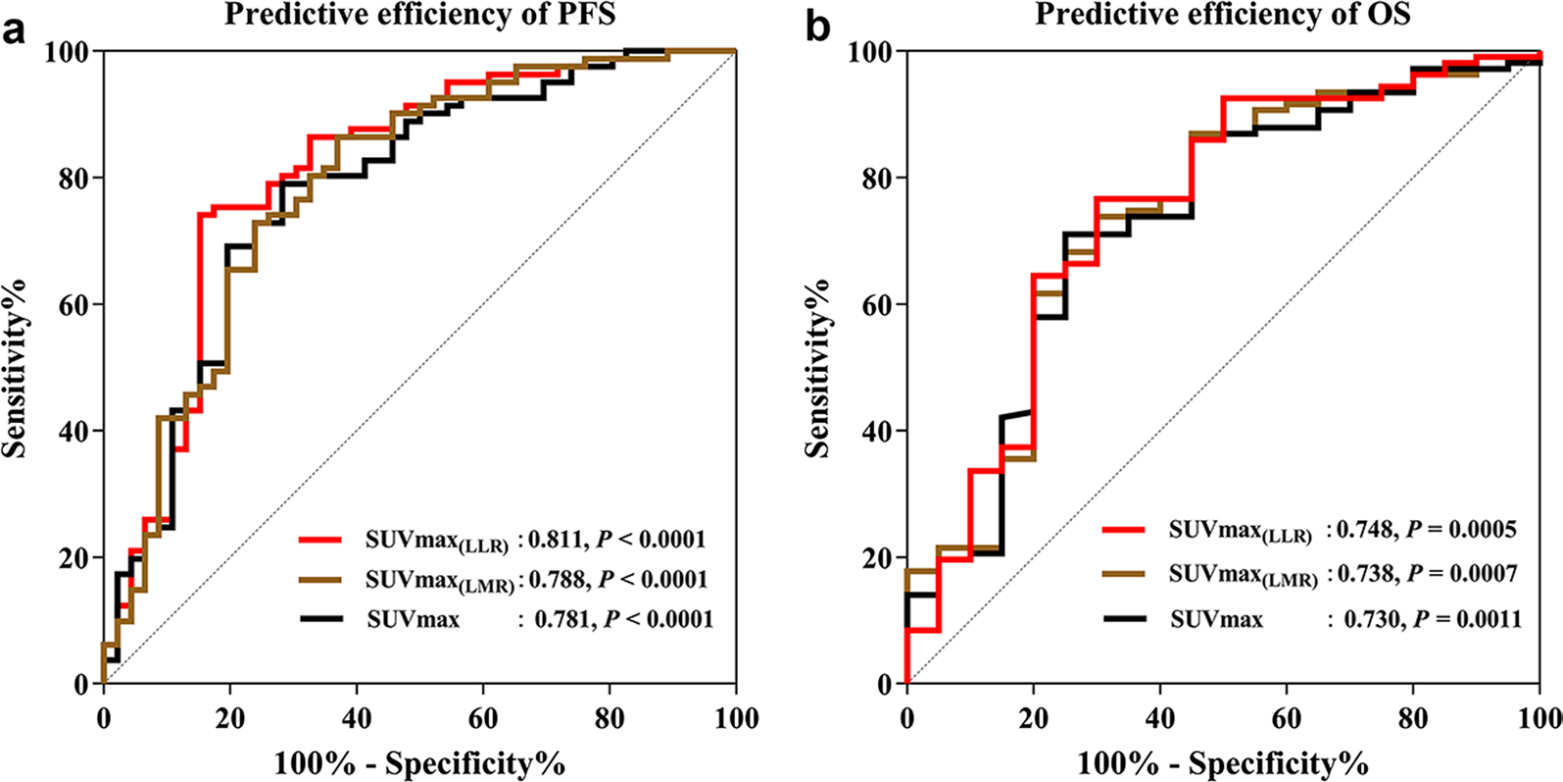
Receiver operating characteristic curve analysis of I-PET-derived parameters to predict disease progression (**a**); areas under the curve were 0.811 for ΔSUVmax_(LLR)_ (*p* < 0.0001), 0.788 for ΔSUVmax_(LMR)_ (*p* < 0.0001) and 0.781 for ΔSUVmax (*p* < 0.0001), respectively. Receiver operating characteristic curve analysis of I-PET-derived parameters to predict disease death (**b**); areas under the curve were 0.748 for ΔSUVmax_(LLR)_ (*p* = 0.0005), 0.738 for ΔSUVmax_(LMR)_ (*p* = 0.0005) and 0.730 for ΔSUVmax (*p* = 0.0011), respectively

First, areas under the curve of ΔSUVmax^1^, ΔSUVmax_(LMR)_^1^ and ΔSUVmax_(LLR)_^1^ to predict disease progression were 0.781, 0.788 and 0.811, and areas under the curve of ΔSUVmax^1^, ΔSUVmax_(LMR)_^1^and ΔSUVmax_(LLR)_^1^ to predict death were 0.730, 0.738 and 0.748, respectively (*p* < 0.05, Fig. 2). This showed that ΔSUVmax_(LLR)_ has the highest prediction performance for PFS and OS. So, in this study, SUVmax_(LLR)_ was used instead of SUVmax.

**Fig. 3:**
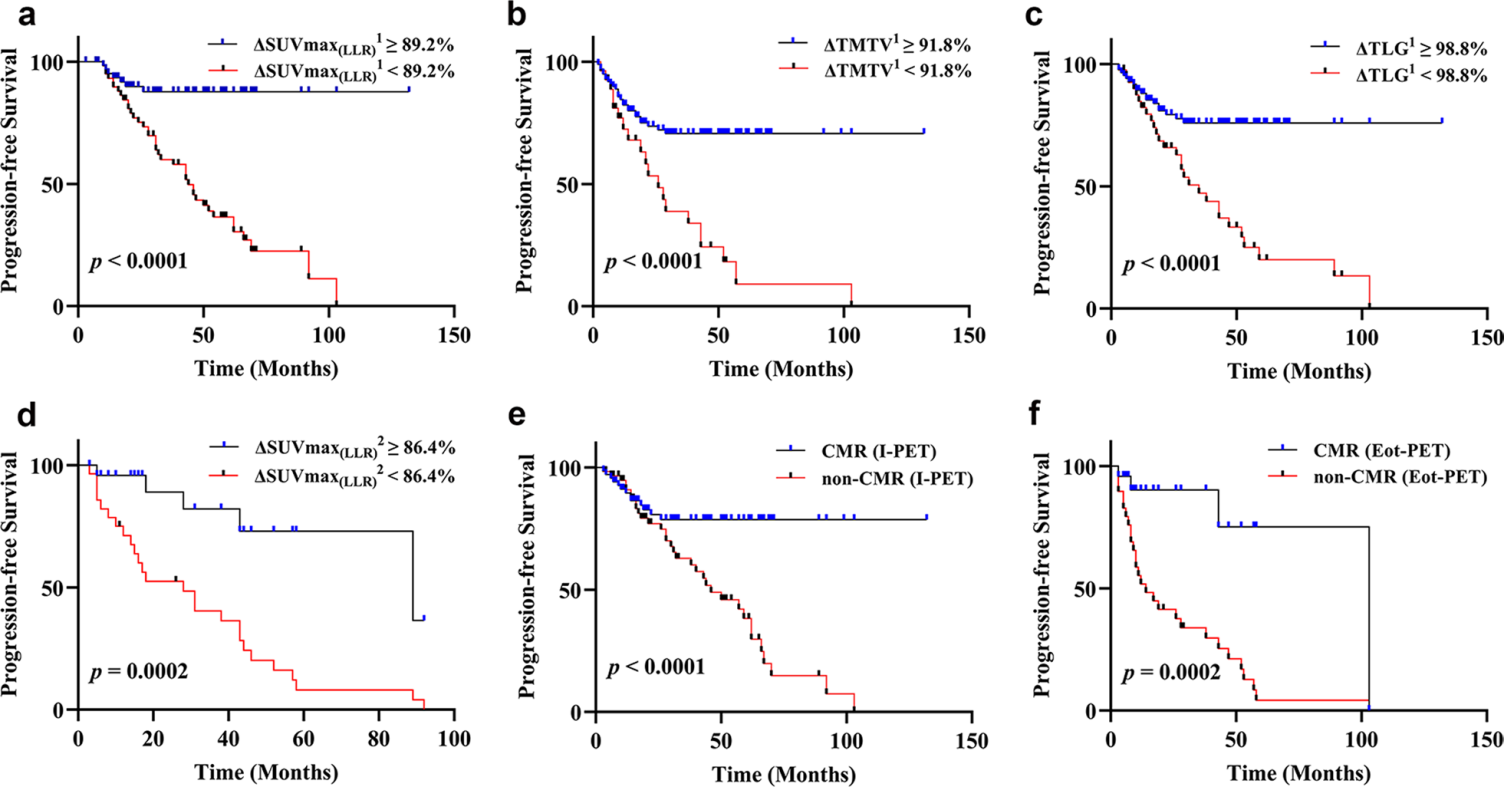
The Kaplan–Meier curve of PFS based on ΔSUVmax_(LLR)_^1^ (**a**, *p* < 0.05), ΔTMTV^1^ (**b**,* p* < 0.05), ΔTLG^1^ (**c**,* p* < 0.05), ΔSUVmax_(LLR)_^2^ (**d**, *p* < 0.05) and treatment response at I-PET(**e**, *p* < 0.05) and Eot-PET (**f**, *p* < 0.05), respectively

Univariate cox regression analysis of PFS showed ΔSUVmax_(LLR)_^1^, ΔTMTV^1^ and ΔTLG^1^ were significantly associated with PFS (*p* < 0.05, Table 3). Multivariate cox regression analysis adjusted the relevant factors and revealed that ΔSUVmax_(LLR)_^1^ (HR 1.024, 95%CI 1.013–1.035, *p* = 0.000), ΔTMTV^1^ (HR 1.026, 95%CI 1.008–1.044, *p* = 0.004) and ΔTLG^1^ (HR 1.030, 95%CI 1.008–1.053, *p* = 0.008) were independent risk factors for PFS (Table 3). Kaplan–Meier survival analysis presented that patients with ΔSUVmax_(LLR)_^1^ < 89.2% (ROC curve, AUC = 0.811, cutoff value), ΔT-MTV^1^ < 91.8% (ROC curve, AUC = 0.700, cutoff value) and ΔTLG^1^ < 98.8% (ROC curve, AUC = 0.767, cutoff value) were significantly associated with poor prognosis. (*p* < 0.05, Fig. 3).

**Table 3:** Univariate and multivariate cox proportional hazards regression for PFS

	Variables	Univariate analysis			Multivariate analysis		
		*p* value	HR	95% CI	*p* value	HR	95% CI
Clinical characteristics	COO subtypes Age Gender Ann Arbor stage IPI LDH β2-MG Ferritin	0.458 0.112 0.559 0.004** 0.006** 0.843 0.060 0.782	0.776 1.020 1.806 3.070 2.460 1.000 1.043 1.000	0.398–1.516 0.995–1.045 0.394–3.493 1.430–6.593 1.292–4.683 1.000–1.000 0.998–1.090 1.000–1.000			
Semi-quantitative parameters	ΔSUVmax_(LLR)_^1^ ΔTMTV^1^ ΔTLG^1^	0.000*** 0.000*** 0.000***	1.030 1.032 1.039	1.021–1.039 1.015–1.048 1.023–1.056	0.000*** 0.004** 0.008**	1.024 1.026 1.030	1.013–1.035 1.008–1.044 1.008–1.053
	ΔSUVmax_(LLR)_^2^	0.000***	1.007	1.003–1.011			
	ΔTMTV^2^	0.525	1.000	0.999–1.001			
	ΔTLG^2^	0.675	1.000	1.000–1.000			
I-VA	CMR or non-CMR	0.000***	4.163	2.184–7.936			
E-VA	CMR or non-CMR	0.000***	10.876	3.765–31.495			

^***^p < 0.001, **p < 0.01, *p < 0.05

*PFS* progression-free survival, *HR* Hazard Ratio, *CI* confidence interval, *COO* cell of origin, *IPI* international prognostic index, *LDH* lactate dehydrogenase, *β2-MG* β2-Microglobulin, *SUVmax* maximum standardized uptake value, *LLR* lesion-to-liver ratio calculated as SUVmax of the residual divided by SUVmax of the liver, *TMTV* total metabolic tumor volume, *TLG* total lesion glycolysis, *VA* visual analysis, *CMR* complete metabolic response, *I* interim, *E* end of treatment, *1* interim compared to baseline, *2* end of treatment compared to baseline

Univariate cox regression analysis of OS showed that ΔSUVmax_(LLR)_^1^, ΔTLG^1^ were significantly associated with OS (*p* < 0.05, Table 4). Multivariate cox regression analysis adjusted the relevant factors and revealed that ΔSUVmax_(LLR)_^1^ (HR 1.009, 95%CI 1.001–1.017, *p* = 0.030), ΔTLG^1^ (HR 1.030, 95%CI 1.006–1.055, *p* = 0.015) were also independent risk factors for OS (Table 4). Kaplan–Meier survival analysis presented that patients with ΔSUVmax_(LLR)_^1^ < 83.2% (ROC curve, AUC = 0.775, cutoff value) and ΔTLG^1^ < 97.6% (ROC curve, AUC = 0.739, cutoff value) were significantly associated with poor survival (*p* < 0.05, Fig. 3).

**Table 4:** Univariate and multivariate cox proportional hazards regression for OS

	Variables	Univariate analysis			Multivariate analysis		
		*P* value	HR	95% CI	*P* value	HR	95% CI
Clinical characteristics	COO subtypes Age Gender Ann Arbor stage IPI LDH β2-MG Ferritin	0.565 0.067 0.516 0.154 0.103 0.838 0.937 0.538	0.682 3.296 1.341 3.036 2.940 1.000 1.000 1.000	0.186–2.506 1.000–9.879 0.553–3.256 0.660–13.962 0.805–10.739 0.999–1.002 1.000–1.000 1.000–1.000			
Semi-quantitative parameters	ΔSUVmax_(LLR)_^1^ ΔTMTV^1^ ΔTLG^1^ ΔSUVmax_(LLR)_^2^ ΔTMTV^2^ ΔTLG^2^	0.005** 0.084 0.001** 0.009** 0.016* 0.080	1.011 1.026 1.038 1.011 1.001 1.000	1.003–1.018 0.997–1.056 1.016–1.016 1.003–1.019 1.000–1.002 1.000–1.000	0.030* 0.015* 0.165 0.519	1.009 1.030 1.008 1.000	1.001–1.017 1.006–1.055 0.997–1.020 0.999–1.002
I-VA E-VA	CMR or non-CMR CMR or non-CMR	0.005** 0.021*	4.288 6.127	1.554–11.838 1.319–28.470			

^***^p < 0.001, **p < 0.01, *p < 0.05

*OS overall survival*, *HR* Hazard Ratio, *CI* confidence interval, *COO* cell of origin, *IPI* international prognostic index, *LDH* lactate dehydrogenase, *β2-MG* β2-Microglobulin, *SUVmax* maximum standardized uptake value, *LLR* lesion-to-liver ratio calculated as SUVmax of the residual divided by SUVmax of the liver, *TMTV* total metabolic tumor volume, *TLG* total lesion glycolysis, *VA* visual analysis, *CMR* complete metabolic response, *I* interim, *E* end of treatment, *1* interim compared to baseline, *2* end of treatment compared to baseline

#### End-of-treatment PET/CT(Eot-PET)

Univariate cox regression analysis of OS showed that ΔSUVmax_(LLR)_^2^ was significantly associated with PFS (*p* < 0.05, Table 3). Kaplan–Meier survival analysis presented that patients with ΔSUVmax_(LLR)_^2^ < 86.4%(ROC curve, AUC = 0.928, cutoff value) was significantly associated with poor prognosis. (*p* < 0.05, Fig. 3). Univariate cox regression analysis of OS showed that all semi-quantitative parameters of Eot-PET had no significant difference in poor survival. (p > 0.05, Table 4).

### Visual analysis

**Fig.4:**
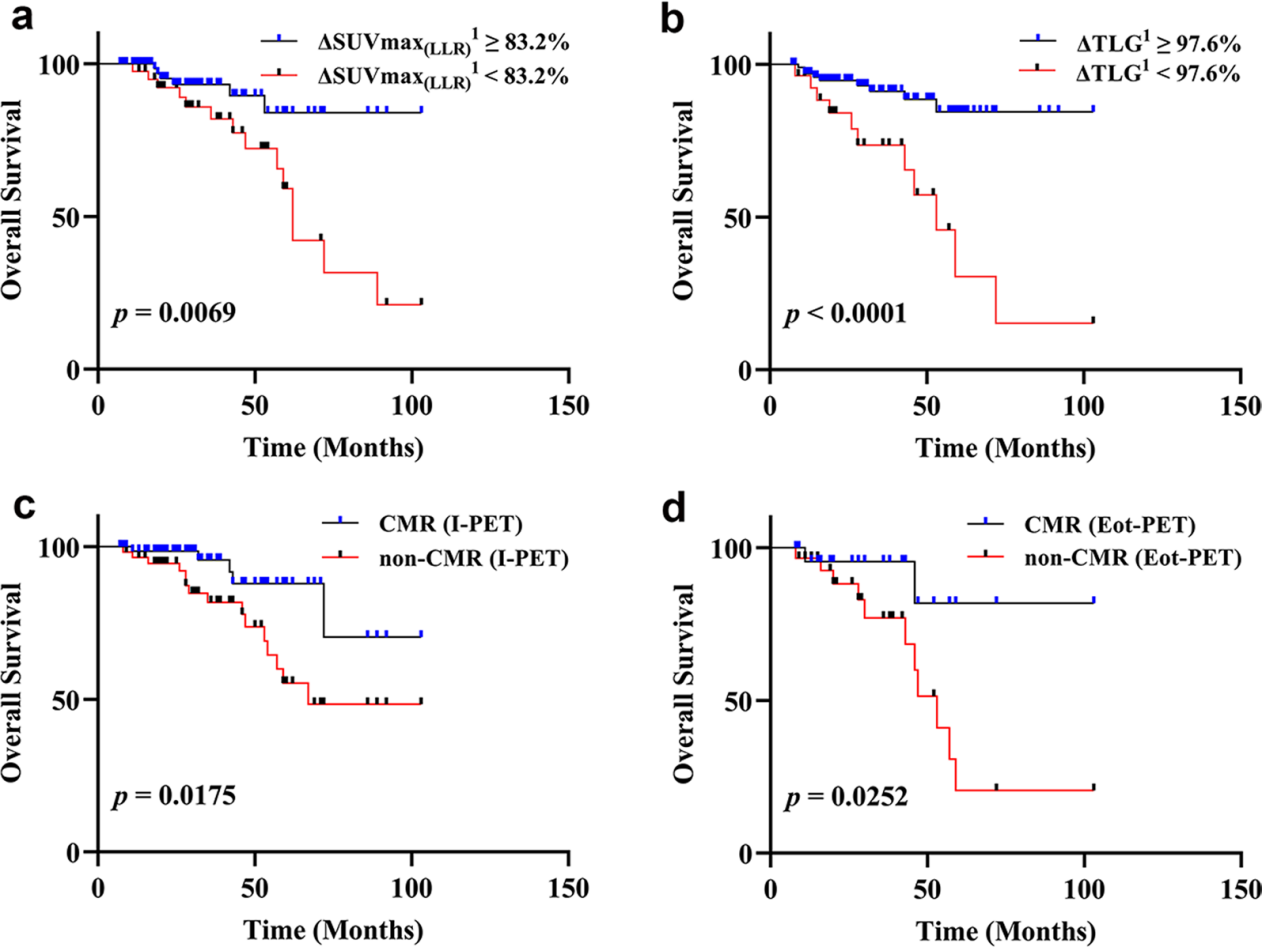
The Kaplan–Meier curve of OS based on ΔSUVmax_(LLR)_^1^ (**a**, *p* < 0.05), ΔTLG^q^ (**b**,* p* < 0.05), treatment response at I-PET (**c**,* p* < 0.05) and Eot-PET (**d**, *p* < 0.05)), respectively

Among the patients with 127 DLBCL, in the follow-up of 81 patients without progression, the proportion of I-PET evaluation of non-CMR was 30.9%, and the proportion of 46 progression patients was non-CMR (31.7%). Fifty-three cases were examined by Eot-PET and 45.3% of them presented with CMR after end of first-line treatment. There was a statistically significant difference between the late progression of DLBCL patients and the I-PET evaluation of CMR. Univariate cox regression analysis of PFS and OS showed that CMR or not assessed by I-PET and Eot-PET were significantly associated with PFS (*p* < 0.05, Tables 3 and 4). Kaplan–Meier survival analysis presented that patients with non-CMR were significantly associated with poor prognosis (*p* < 0.05, Figs. 3 and 4) (*p* < 0.05, Table 5).

**Table 5:** The predictive performance of I-PET/CT parameters in predicting disease progression

	Sensitivity	Specificity	PPV	NPV	Accuracy
VA	71.7	69.1	56.9	81.6	70.1
ΔSUVmax_(LLR)_^1^	84.8	74.1	65.0	89.6	78.0
ΔSUVmax_(LLR)_^1^ + VA	67.4	84.1	70.5	81.9	78.0
ΔTMTV^1^	43.5	91.4	74.1	74.0	74.0
ΔTMTV^1^ + VA	41.3	97.5	90.5	74.5	77.2
ΔTLG^1^	60.9	84.0	68.3	79.1	75.6
ΔTLG^1^ + VA	58.7	92.6	81.8	79.8	80.3

*PPV* Positive Predictive Value, *NPV* Negative Predictive Value, *VA* Visual analysis, *SUVmax* maximum standardized uptake value, *LLR* lesion-to-liver ratio calculated as SUVmax of the residual divided by SUVmax of the liver, *TMTV* total metabolic tumor volume, *TLG* total lesion glycolysis,* 1* interim compared to baseline

### Diagnostic performance

In terms of predicting recurrence by I-PET, the combination of visual and semi-quantitative parameters showed higher PPV and specificity than a single index. Through the above statistical analysis, the value of I-PET in predicting the prognosis of DLBCL is higher. Therefore, we used I-PET parameters for further analysis. All diagnostic performance values are detailed in Table 5. According to the findings of our study, the combination of visual and semi-quantitative analysis may be considerably more reliable for early prediction of DLBCL chemotherapeutic response and final results.

## Discussion

Nowadays, the prognostic evaluation of DLBCL remains critical issues in clinical practice. Although the cure rate of DLBCL has been significantly improved in the era of rituximab, some patients still develop recurrent/refractory DLBCL (R/R DLBCL) and the prognosis is poor after salvage treatment. Therefore, it is necessary to identify and give active treatment as soon as possible, rather than just waiting for the treatment to fail or relapse. Methods for predicting the response and final outcome of DLBCL chemotherapy must be able to distinguish between patients who will be cured if the standard regimen is continued and those who will not achieve lasting remission unless intensive therapy is implemented. Based on the above, the aim of this study was to evaluate how to find DLBCL patients with poor prognosis timely and accurately.

At present, relevant studies have shown that, compared with pre-treatment indicators such as the International Prognostic Index (IPI) of diffuse large B cell Lymphoma [29] or the International Prognostic Score (IPS) of Hodgkin’s Disease [30], PET/CT-related imaging data in the middle stage of chemotherapy (3–4 cycles) have been proved to be a more ideal and independent index for early treatment to predict the efficacy and progression-free survival of patients[31–34]. In the study on the timing of I-PET in 1692 patients with DLBCL [34], it is suggested that PET/CT examination after 4 cycles of treatment has higher discrimination than 2 cycles, which may be the starting point of the new treatment. Poor response at I-PET after 4 cycles using ΔSUVmax response criteria may work best for randomized trials evaluating new therapy regimens. Nonetheless, some studies reported that increased false positive rate of I-PET [35, 36], a concern with the long half-life and unique mechanisms of cytotoxicity of rituximab, resulted in the failure of predicting outcomes. Thus, use of a standard operating protocol and harmonized criteria for prediction of outcomes based on I-PET/CT and Eot-PET/CT findings in homogenous populations has distinct advantages. Our study followed the standard, all the DLBCL patients who received Eot-PET/CT also received baseline and interim PET/CT assessment, but some of the patients after interim PET/CT did not continue PET/CT assessment. That’s why, only 53 patients have an Eot-PET/CT. In our study, both semi-quantitative and visual analyses showed that compared with Eot-PET/CT, I-PET/CT had higher prognostic value in patients with DLBCL. It can be said that poor response at I-PET after 3–4 cycles may work best for randomized trials evaluating new therapy regimens.

The next problem that needed to be solved was how to find patients with poor prognosis more accurately in the middle stage of chemotherapy, that is, what response criteria at I-PET/CT do we need to use to accurately predict the prognosis?

D-5PS is a visual evaluation scale, it is the most commonly used clinical PET/CT analysis method [37, 38], and it has a certain value in predicting the prognosis of DLBCL [38, 39]. Numerous studies have shown that those with an early complete metabolic response (I-PET) have event-free survivals in excess of 80% [20, 40]. Our study found that these early metabolic responders had excellent survival outcomes, with a 2-year PFS of 84.6% and a 2-year OS of 96.0%. Second, only approximately 5.1% (13/127) of patients exhibited rapid disease progression and were considered as SD/PD at I-PET. The survival outcomes for these patients were poor, with median PFS and OS of just 6 months and 32 months, respectively. Multivariable analyses further confirmed that both I-PET and Eot-PET positivity were independently associated with patient prognosis. Notably, the prognostic of D-5PS is under debate due to its low PPV [41, 42]. In recent years, the potential value of semi-quantitative parameters of ^18^F-FDG PET/CT in evaluating the prognosis of DLBCL has gradually become a research hotspot [43]. Semi-quantitative parameters can better reflect the dynamic process of tumor, improve the performance of prognosis prediction, reduce false positive and have higher diagnostic consistency among different observers [44]. Many authors demonstrated that semi-quantitative analysis could outperform the visual analysis [33, 45]. Some studies showed better reproducibility, accuracy and PPV of SUVmax-liver-based interpretation than that with use of the D-5PS and SUVmax scale criteria [46, 47]. Our study showed that the area under ROC (AUC) of SUVmax_(LLR)_ is higher than that of SUVmax and SUVmax_(LMR)_, in the survival analysis of this study, we use SUVmax_(LLR)_ instead of SUVmax. Previous studies reported that the best critical value of ΔSUVmax is 66–81.5%[5, 48]. In this study, the best critical value for SUVmax_(LLR)_ at I-PET/CT to evaluate PFS was 89.2% and the best critical value for OS was 83.2%. The reason why it was different from other studies may be that SUVmax was based on liver correction.

SUVmax only reflects the most obvious metabolic activity of a tumor nodule and does not represent the metabolic activity of all tumors. For patients with heavy tumor load, its prognostic value is limited [49] and SUVmax is also affected by local inflammation, serum glucose levels and other confounding factors. Both MTV and TLG can provide certain reference value for the prediction of short-term curative effect and long-term prognosis of tumors. MTV can better estimate the tumor burden. TLG is the product of SUVmean and MTV, which represents the metabolic burden of the tumor, which depends on the metabolic volume and glucose utilization of the tumor. At present, the role of MTV and TLG in predicting the efficacy and evaluating the prognosis of patients with solid tumor has gradually become a research hotspot, including non-small cell lung cancer [50], lymphoma, and so on. In the present study, MTV and TLG were shown to be significantly associated with PFS and OS in DLBCL [10]. In our study, MTV and TLG showed prognostic value to some extent. ΔTMTV^1^ < 91.8%, ΔTLG^1^ < 98.8% were significantly associated with poor prognosis, and ΔTLG^1^ < 97.6% was significantly associated with poor survival.

**Fig. 5:**
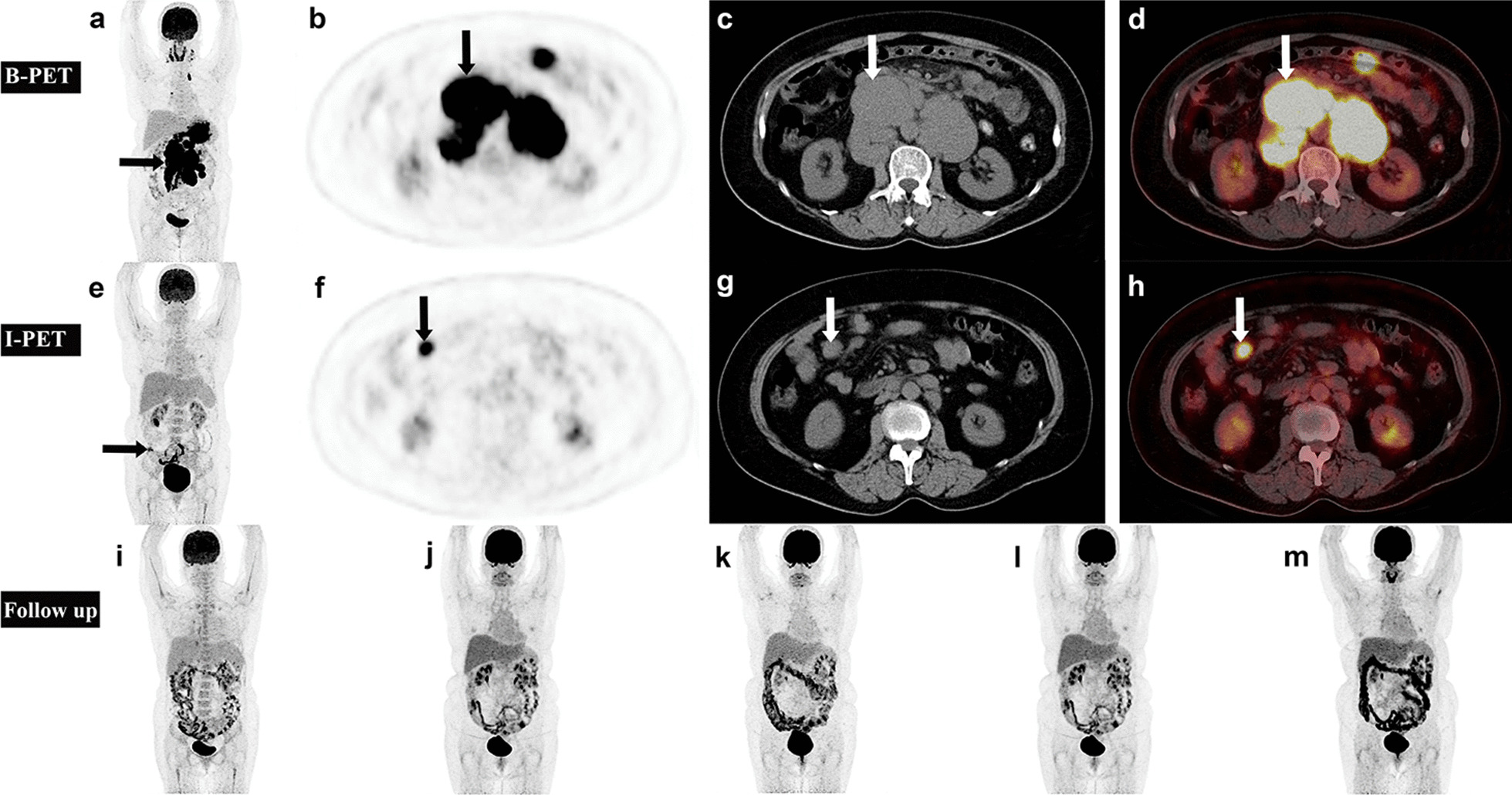
An example was evaluated by visual and semi-quantitative parameters of I-PET4 as a poor prognosis group, and the prognosis was improved by intensive treatment. A 57-year-old woman diagnosed DLBCL by puncture for more than 2 weeks. Baseline ^18^F-FDG PET/CT showed intense glucose uptake in multiple systemic lymph nodes (mainly celiac lymph nodes, large arrow) on MIP (**a**). Axial images of abdomen region (**b** PET; **c** CT; d fusion) revealed an enlarged lymph nodes of size 60 × 50 mm and SUVmax(LLR) of 9.7 on the right side of the abdominal aorta (arrow). Interim ^18^F-FDG PET/CT showed intense glucose uptake in the right side of the abdominal cavity on MIP (**e**). Axial images of the abdomen region (**f** PET; **g** CT; h fusion) revealed a lymph node of 22 × 19 mm and SUVmax_(LLR)_ of 5.9 in the mesentery (arrow). Evaluation of curative effect: visual analysis is PMR, ΔSUVmax_(LLR)_ = 39.47%. After interim evaluation, the patient received autologous stem-cell transplantation (ASCT) and rituximab maintenance therapy. During the follow-up, the abdominal lesions disappeared and the prognosis was good. 18F-FDG PET/CT shows that the patient is in CMR state on MIP (i-m)

**Fig. 6:**
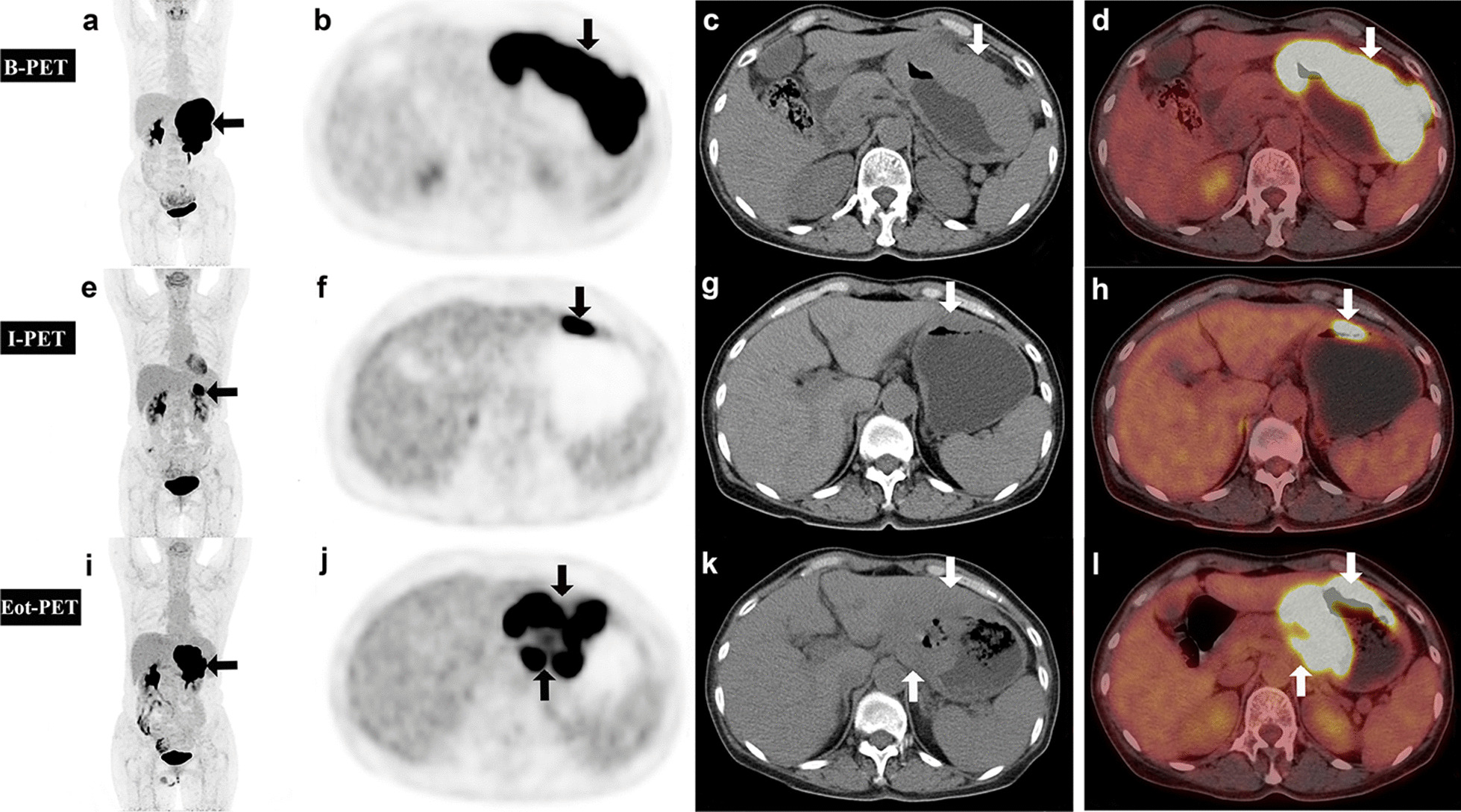
An example was evaluated by visual and semi-quantitative parameters of I-PET/CT4 as a poor prognosis group, and the prognosis was poor without further intensive treatment or change of treatment regimen. A 62-year-old woman diagnosed with DLBCL by gastroscopy for 1 week. Baseline ^18^F-FDG PET/CT showed intense glucose uptake (arrow) in the whole gastric wall on MIP (**a**). Axial images of abdomen region (**b** PET; **c** CT; **d** fusion) revealed diffuse thickening of whole gastric wall and SUVmax_(LLR)_ of 16.6 (arrow). Interim ^18^F-FDG PET/CT showed intense glucose uptake in in local gastric wall on MIP (**e**). Axial images of abdomen region (**f** PET; **g** CT; h fusion) revealed thickening of gastric wall on the side of great curvature of gastric body and SUVmax_(LLR)_ of 10.0. Evaluation of curative effect: visual analysis is SMD, ΔSUVmax _(LLR)_ = 39.87%. After interim evaluation, R-CHOP continued to be treated for 4 courses. End-of-treatment ^18^F-FDG PET/CT showed intense glucose uptake (arrow) in the most of gastric wall on MIP (**i**). Axial images of abdomen region (**j** PET; **k** CT; l fusion) revealed thickening of the gastric body and horn with pancreatic invasion and SUVmax_(LLR)_ of 13.8. The patient died 10 months later, and the overall survival time was less than 2 years

Most of the studies on the effect of I-PET on prognosis are that the NPV value has been very high, but the PPV value varies greatly, about 40–65% [22, 35, 40, 51, 52]. Therefore, it is necessary to improve the stratification of prognosis. It is worth emphasizing that our findings are not only that, the combination of visual and semi-quantitative parameters showed higher PPV and specificity than a single index. The risk of PFS and OS shortening in patients with DLBCL below the threshold was increased due to poor mid-term evaluation and semi-quantitative analysis. Therefore, the intensification of treatment regimens based upon I-PET positivity may reduce the risk of relapse and death in patient. However, it is necessary to combine visual and semi-quantitative threshold comprehensive evaluation, otherwise it would likely expose many patients to the risk of unnecessary treatment. Our study shows that combined visual and semi-quantitative parameters evaluation can improve the ability of I-PET/CT to predict prognosis, which was superior to a single index and also was superior to current clinical indices of risk (IPI). As shown in Table 5, combined visual and semi-quantitative analysis at I-PET can more effectively distinguish patients with poor prognosis in order to strengthen treatment as soon as possible (Figs. 5 and 6). Compared with other studies, the PPV of this study is relatively high, which provides a more reliable basis for early clinical selection of DLBCL patients who need intensive treatment to improve the prognosis.

Clearly, there were some limitations and shortcomings in this study, which was limited by its single-center and retrospective nature; accordingly, there was a possibility of selection bias. In our study, among the 127 patients, only 53 patients underwent Eot-PET/CT, there may be some differences in the results of Eot-PET/CT survival analysis among the same population. Besides, among the patients in our group, a small number of patients were tested for gene, and the effect of gene mutation on prognosis was not considered. In future, the prognostic power of the evaluation system for B-PET/CT, I-PET and Eot-PET should be further tested with prospective research in a larger patient population.

## Conclusion

Semi-quantitative parameters of ^18^F-FDG PET/CT have certain value in predicting the prognosis of DLBCL at baseline, interim and end of first-line treatment, and SUVmax_(LLR)_ is relatively more efficient than SUVmax in predicting prognosis. Three to four cycles of R-CHOP treatment can be used as a time point for early prediction of R/R DLBCL, and the combination of visual analysis and semi-quantitative parameters can improve the accuracy of predicting prognosis, thus helping patients in need of early intensive treatment for more accurate screening and helping clinicians to choose a more appropriate treatment plan as soon as possible.

### Supplementary Information

**Additional file 1. Fig. S1.** Illustrative diagram depicting the method used for identifying DLBCL lesions and calculate TMTV and TLG. Mip image of the sample patient (a). Identify DLBCL lesions (SUVmax ≥ 2.5, red areas, b), exclude physiological uptake (blue areas, b); TMTV, TLG calculated automatically (c).

## Data Availability

The datasets generated during and/or analyzed during the current study are available from the corresponding author on reasonable request.

## Abbreviations

B: Baseline
I: Interim
Eot: End of treatment
DLBCL: Diffuse large B cell lymphoma
SUVmax_(LLR)_: Maximum standardized uptake value of lesion-to-liver ratio
SUVmax_(LMR)_: Maximum standardized uptake value of lesion-to-mediastinum ratio
TMTV: Total metabolic tumor volume
TLG: Total metabolic tumor volume
D-5PS: Deauville 5-point scale
VA: Visual analysis
PFS: Progression-free survival
OS: Overall survival
CAR-T: Chimeric antigen receptor T cell
NHL: Non-Hodgkin lymphoma
ASCT: Autologous stem-cell transplantation
IPI: International Prognostic Indices
LDH: Lactate dehydrogenase
β2-MG: Beta-2-microglobulin
R/R: Recurrent/refractory
