# Supplementary figures and images for: Evaluation of therapeutic effect and prognostic value of 18F-FDG PET/CT in different treatment nodes of DLBCL patients
Source: EJNMMI Res. 2024 Feb 19;14:20. doi: 10.1186/s13550-024-01074-w (PMC10876506; doi:10.1186/s13550-024-01074-w)

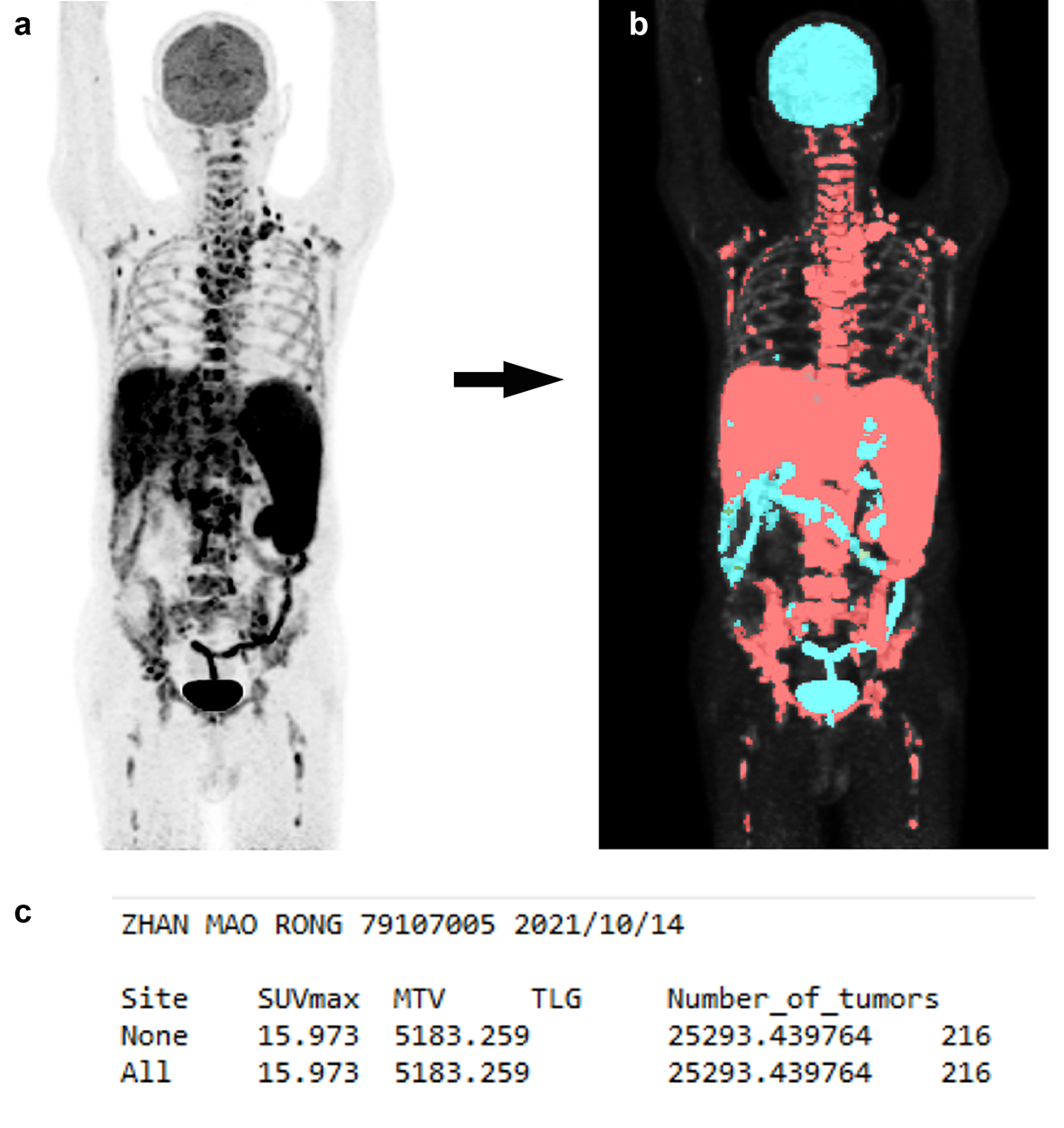

Supplement: Supplementary file 1 — Additional file 1. Fig. S1. Illustrative diagram depicting the method used for identifying DLBCL lesions and calculate TMTV and TLG. Mip image of the sample patient (a). Identify DLBCL lesions (SUVmax ≥ 2.5, red areas, b), exclude physiological uptake (blue areas, b); TMTV, TLG calculated automatically (c). [file 13550_2024_1074_MOESM1_ESM.tif]
